# The complete genome sequence of the algicidal bacterium *Bacillus subtilis* strain JA and the use of quorum sensing to evaluate its antialgal ability

**DOI:** 10.1016/j.btre.2020.e00421

**Published:** 2020-01-09

**Authors:** Sheng-Jie Zhang, Xiao-Peng Du, Jian-Ming Zhu, Chen-Xu Meng, Jin Zhou, Ping Zuo

**Affiliations:** a The Shenzhen International Graduate School, Tsinghua University, Shenzhen, 518055, Guangdong Province, PR China; b School of Environment, Harbin Institute of Technology, Harbin, 150090, Heilongjiang Province, PR China; c Second Institute of Oceanography, Ministry of Natural Resources, Hanzhou, 310000, Zhejiang Province, PR China; d The School of Geography and Ocean Science, Nanjing University, Nanjing, 210093, Jiangsu Province, PR China

**Keywords:** *B. subtilis* JA, Quorum sensing, Whole-genome sequencing, Antialgal agent, Harmful algal blooms

## Abstract

We describe the isolation of *Bacillus subtilis* strain JA and demonstrate that this bacterium exhibited strong algicidal effects on the algae *Alexandrium minutum* with an inhibition rate exceeding 80 % within 48 h*. B. subtilis* JA significantly reduced the photosynthetic efficiency of *A. minutum* and caused extensive morphological damage to the algae. Genomic analysis of *B. subtilis* JA demonstrated that a putative AI-2 type quorum sensing (QS) gene (*LuxS*) is present in its genome cluster, which is regulate pheromone biosynthesis. Interestingly, the exogenous addition of a QS-oligopeptide (ComX-pheromone) improved the algicidal efficiency of *B. subtilis* JA, thus indicating that the algicidal activity of this bacterium is potentially regulated by QS. Collectively, our data describe a potential antialgal bacterium and speculated that its behavior can be modulated by QS signal. *B. subtilis* JA may therefore represent a valuable tool for the development of novel chemical-ecological methods with which to control harmful algae.

## Highlights

• *B. subtilis* strain JA exhibit strong algicidal effects on algae with the inhibition rate exceeding 80 % within 48 h.
• The algicidal activity is regulated by AI-2 type quorum sensing.
• The complete genome information is provided for developing novel chemical-ecological methods to control harmful algae.

## 1 Introduction

Harmful algal blooms (HABs) are caused by the rapid proliferation of algae in response to eutrophication and pose a significant threat to both the environment and human health [1]. Over recent years, HABs have become a frequent occurrence alongside environmental pollution and climate change [2]. HABs can intoxicate humans via the ingestion of contaminated seafood, reduce stocks of wild or cultured fish, and impair tourism [3]. Under such circumstances, there is an urgent need to develop methods with which to control algal expansion. Previous work has described the application of both physical and chemical methods to prevent or remove HABs [4]. However, physical and chemical methods are not optimal choices for controlling HABs because such approaches are costly, cannot be applied on a large-scale and, more importantly, because they can give rise to secondary pollution [5]. Consequently, biological methods are regarded as more promising approaches with which to regulate HABs, largely because such methods may be more targeted and native to aquatic environments and better biosafety for aquatic organisms [6,7]. One such biological method is the use of algicidal bacteria; these have been shown to play an important role in lysing algal cells in aquatic ecosystems [8,9]. Most of these bacteria have an algicidal effect that is relatively species-specific. For example, Li et al. [8] isolated *Bacillus* sp. strain Lzh-5 and demonstrated that compounds derived from this strain exhibited strong algicidal activity against *Microcystis aeruginosa*. [10,11]) also reported that *Enterobacter cloacae* NP23 and *Gibberella moniliformis* AN11 have high speciﬁcity to inhibition *Chlorella pyrenoidosa* by disrupt host oxidative balance (i.e. inhibition of antioxidase activities).

The ecological behavior of bacteria can be modulated by chemical signals, including quorum sensing (QS). Previous literature has reported that QS is able to influence various microbial behaviors, including the production of secondary metabolites and microbial virulence factors, the formation of biofilms, cell motility and conjugation, and antibiotic resistance ([[12], [13], [14]]). The extent to which QS can modulate such behaviors, however, tends to be species-specific. The best-studied QS protein in Gram-negative bacteria is N-acyl homoserine lactone (AHL). Unlike their Gram-negative counterparts, Gram-positive bacteria generally use oligopeptides (for example, autoinducer-2, AI-2) as signaling molecules in QS-controlled gene expression systems. The precursor of AI-2 is (*S*)-4,5-dihydroxy-2,3-pentanedione, which undergoes catalysis by S-ribosylhomocysteine lyase (*LuxS*), an enzyme that plays a key role in methionine and cysteine metabolic pathways. Previous research showed that AI-2 is produced by a wide range of bacteria, including *Bacillus* spp.. Furthermore, AI-2 signaling has been considered as a possible strategy with which to modulate the behavior of Gram-positive bacteria in specialized microecosystems [15].

*Bacillus subtilis* is a typical Gram-positive bacteria and utilized quorum-sensing pheromones as signal molecules to communicate among individual cells to regulate their activities as a group through a cell density-sensing mechanism. The competence pheromone was first described genetically in *B. subtilis* 168 [16]. In this bacterium, possesses a regulatory system encoded on the ComQXPA gene cluster to mediate quorum-sensing responses [17]. ComQ is involved in the biosynthesis of an oligopeptide-type bacterial pheromone called the ComX pheromone. The ComX pheromone is a signaling oligopeptide that stimulates natural genetic competence controlled by quorum sensing in *Bacillus subtilis* [18]. Previously, Esmaeilishirazifard et al. [19] show that ComX filtered extracts from cultures of *B. licheniformis* can significant inhibit the growth of fungi (*A. flavus* NRRL 3357 and ESP 15).

Some researchers have reported that the algicidal activity of *B. subtilis* is related by QS signals [20]. We also observed this phenomenon and screened a potential algicidal bacterium (*Bacillus subtilis* strain JA) isolated from a phycosphere environment. Although a phenotype was observed, the ability of such bacteria to exert antialgal effects has not been elucidated at the molecular level using genomic approaches. It is also evident that while the algicidal effects of certain bacteria are believed to regulated by QS, there is no direct evidence at present to support this notion.

In this study, we determined the full genome sequence of *B. subtilis* strain JA by next-generation sequencing (NGS) technology. We then identified the genomic features of genes related to AI-2. Finally, we evaluated the algicidal activity of *B. subtilis* strain JA in the presence of an exogenous oligopeptide (ComX pheromone). Collectively, our results provide a deeper insight into the behavior of Gram-positive bacteria in response to QS signaling and will facilitate the development of environmentally friendly methods with which to control HABs.

## 2 Materials and methods

### 2.1 Sampling and the isolation of bacteria

Surface water samples were collected from blooms of *Alexandrium* sp. off the coast of Shenzhen. These samples were then serially diluted (10^−1^ to 10^−7^-fold) with sterilized seawater. A 50 μl aliquot of each dilution was then spread onto 2216E agar plates (BD Diagnostic Systems, Dingguo, China) and then incubated at 30 °C for 48−72 h. After incubation, colonies were randomly selected according to their morphology, size, and color, and then transferred to 1.5 mL microcentrifuge tubes containing 2216E liquid medium; these were then incubated for 24−48 h.

### 2.2 Analysis of algicidal rate

*Alexandrium minutum* was used as the target algae and was cultivated in batch cultures of sterilized f/2 medium at 23 °C, with a 12/12 h light/dark cycle. Illumination intensity was 3000 lx and salinity was 30‰. Prior to the experiments, algae were transferred to new cultures and grown to the logarithmic growth phase.

Isolated bacterial strains were grown in 100 mL of 2216E liquid medium at 30 °C for 48 h. Then, a 1 mL aliquot of each isolate culture was inoculated (in triplicate) into 100 mL of *A. minutum* cultures in the logarithmic-phase. Then, a 1 mL aliquot of 2216E liquid medium was added to the algal cultures as a control. During the incubation process, algicidal rate was tested by counting the number of algal cells. Algicidal rate was calculated according to Eq. (1) [9] in which *N_C_* and *N_E_* represent the number of algal cells in control and experimental groups, respectively.

(1) $$\text{A}\text{l}\text{g}\text{i}\text{c}\text{i}\text{d}\text{a}\text{l}\;\text{r}\text{a}\text{t}\text{e}\;\left(\text{\%}\right)=\frac{N_{C}-N_{E}}{N_{C}}\times 100\%$$

The algicidal rate of samples from the *A. minutum* inoculation system was determined every 12 h. Algal cells were first fixed in Lugol’s iodine reagent and then counted under a light microscope (Zeiss, Germany). All experiments were repeated with three biological replicates. The control group consisted of normal algae grown in sterile 2216E medium or sterile f/2 medium to avoid the influence of different media. Algicidal rate was determined to estimate the algicidal activity of the bacterial strain. We also determined the Chlorophyll *a* content and photochemical efﬁciency (Fv/Fm value) using a PhytoPan ﬂuorescence monitoring system (Effeltrich, Germany). *B. subtilis* strain JA showed the highest algicidal rate and was thus analyzed further in subsequent experiments.

### 2.3 Detection of the AI-2 bioluminescence in *B. subtilis* strain JA

We carried out an AI-2 bioluminescence assay according to the methods described by Bassler et al. (1997) and Qian et al. (2015). In brief, *B. subtilis* strain JA was grown in 2216E medium at 30 °C with shaking at 200 rpm. Twenty min after the onset of the stationary phase, the supernatant was harvested by centrifugation (15 min, 4000 × g) and passed through a 0.22-μm membrane ﬁlter. We used *Vibrio harveyi* TL26 as a reporter strain as these bacteria respond only to the presence of exogenous AI-2 following the induction of bioluminescence. The cell-free supernatant of *V. harveyi* BB152 and 2216E medium were used as positive and negative controls, respectively. Prior to assay, strains of *V. harveyi* were grown in 2216E medium at 30 °C with shaking at 150 rpm for 12 h. The culture of *V. harveyi* TL26 was then diluted (1:500) in fresh 2216E medium and distributed in 96-well assay plates (180 μl per well). Afterwards, 20 μl of the cell-free supernatants from *B. subtilis* JA strains, or the positive and negative controls, were added into each well containing the reporter strain. The plate was then placed in a luminometer (Fluostar Galaxy, BMG Labtech, Germany) and AI-2 activity was measured for 12 h at 30 °C. All the experiments were performed in triplicate. Relative AI-2 activity was determined by the following equation: (mean bioluminescence of test sample–mean bioluminescence of 2216E medium)/(mean bioluminescence of BB152 strain supernatant–mean bioluminescence of 2216E medium) × 100 %.

### 2.4 Algicidal activity in the absence or presence of an oligopeptide

In *Bacillus subtilis*, the ComX/P/A signalling pathway is a major quorum response pathway and regulates the development of genetic competence [21]. The ComX pheromone is a farnesylated 10-amino-acid peptide that is secreted and accumulates extracellularly [22]. In order to investigate the role of QS in regulating algicidal ability, we evaluated the effect of adding a standard oligopeptide (ComX pheromone); the methodology for this procedure has been described previously [9]. In brief, 1 × 10^4^ cells/ml of *A.* minutumalgae (in logarithmic growth phase) were inoculated into a 250 ml bottle in sterile f/2 medium. *B. subtilis* JA (1 × 10^7^ cells/mL) was then incubated with the algae in the presence or absence of ComX pheromone (1 mM). The initial incubation time was regarded as 0 h and algicidal rates were determined every 12 h for a total period of 120 h.

### 2.5 Complete genome sequencing and assembly

Genomic DNA was first isolated using a DNA Kit (mBio, USA) and then purified by a DNA extraction kit using methodology described by the manufacturers. The concentration and integrity of the extracted DNA were subsequently analyzed using a Nanodrop (Thermo Scientific, USA) and DNA gel electrophoresis, respectively. Spectrophotometry estimated that the purity of the DNA purity was 1.8 (280/260). The purified DNA was then used for genome sequencing. In brief, a sequencing library for *B. subtilis* JA was prepared using a TruseqDNA sample preparation kit, V2 (Illumina Inc. CA). Whole-genome next-generation sequencing was performed on an Illumina Hiseq 2000 platform by BGI Company. Low-quality reads were filtered out prior to *de novo* genome assembly by SOAP v1.05 to generate 21 contigs and 6 scaffolds. Paired-end relationships were then used to connect the resultant scaffolds [23]. GapCloser was then used to fill in gaps along the assembled genome.

### 2.6 Functional annotation of genome of *B. subtilis* strain JA Functional annotation of genome of *B. Subtilis* strain JA

*In silico* annotation was performed using prodigal (v2.60) software. Open reading frames (ORFs) were then annotated by Blast2GO software in accordance with the National Center for Biotechnology Information (NCBI) database [24]. The annotated information was manually curated prior to categorization into Clusters of Orthologous Groups (COGs). Functional COGs were subsequently identified through homologous comparisons using BLASTp. RNAmmer [25] was used to identify rRNA operons and clustered s-rRNA genes while tRNAscan-SE was used to identify tRNAs [26]. We used the *B. subtilis* strain JA genome as a reference and compared this with the genomic sequence of *B. subtilis* 168, which we obtained from the NCBI database (NC_000964.3). Circos representations were then used to compare the genomes of these two strains of bacteria.

In order to identify putative genes related to AI-2, the ORFs were further searched using the NCBI nucleotide sequence and Uniprot databases to identify the AI-2 encoding-protein and its corresponding responsers (such as ComP/X/A). The protein sequences obtained were then further validated with InterproScan (http://www.ebi.ac.uk/interpro/).

### 2.7 Statistical analysis

Algicidal rates were expressed as mean ± standard deviation. P values < 0.05 or 0.01 were considered to be statistically significant. Data were processed by one-way analysis of variance (ANOVA) using SPSS version 13.0 (SPSS, USA).

## 3 Results and discussion

### 3.1 Identification of *B. subtilis* JA strain and its algicidal activity

**Fig. 1:**
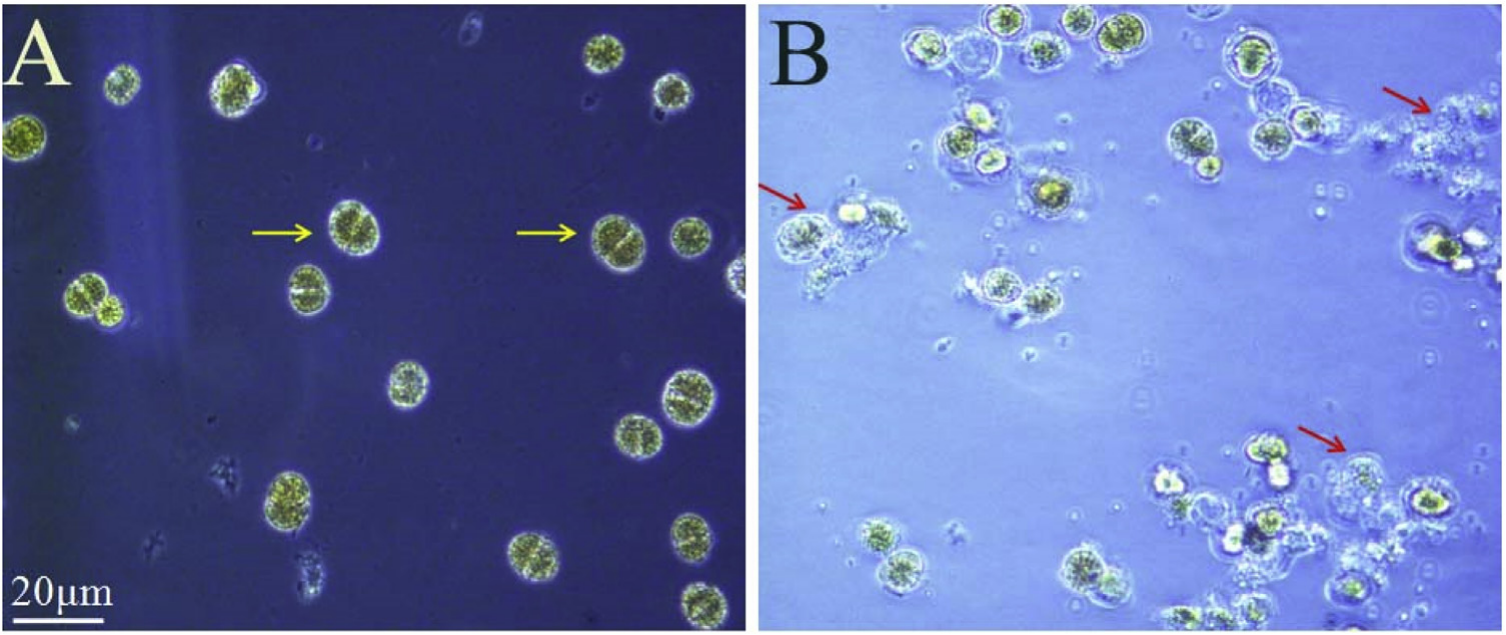
Cellular morphology of algal cells (B) after two days of exposure to strain JA, as compared to those in the control groups (A).

**Fig. 2:**
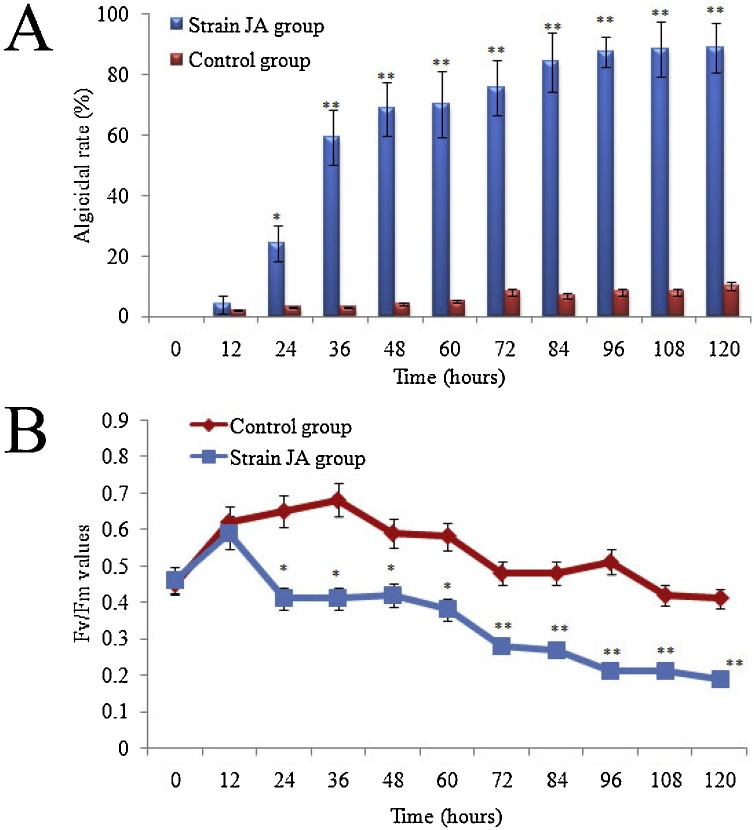
(A) The algicidal rate of *B. subtilis* JA during the culture process. (B) The photosynthetic parameters (Fv/Fm values) of *A. minutum* treated by JA strain, the single and double asterisk represent statistically significant differences at p < 0.05 and p < 0.01 level, respectively. Data represent the mean + SD of triplicate measurements (n = 3).

16S rRNA gene sequence analysis revealed that the JA strain shared 99.9 % similarity with the *B. subtilis* 168 strain. Hence, we provisionally named this strain as *B. subtilis* strain JA. The general features of strain JA are summarized in Table 1. Perhaps the most interesting ecological function of this strain is its algicidal effect on *A. minitum*. *B. subtilis* strain JA clearly exhibited the ability to disrupt the cellular morphology of *A. minitum* over the experimental process (Fig. 1). Control cells showed a low rate of inhibition. In contrast, the algicidal rate in the treatment group was significantly increased following treatment with *B. subtilis* strain JA for 24 h (P < 0.05) (Fig. 2A). In addition, the Fv/Fm value of the algal cells treated by strain JA decreased signiﬁcantly compared with the control group (P < 0.01) (Fig. 2B). The Fv/Fm value reﬂects the potential maximum photosynthetic capacity; this value will decrease when algae suffer from external stress. The reduction of photosynthetic efficiency observed following treatment with strain JA indicated that this bacterium represents a potential algaecide in the future. Algaecides that are derived from environmental microorganisms are very economical due to the low cost of the source material and because they are ecologically friendly [27].

**Table 1:** The general and genome features of *B. subtilis* JA.

Item	Description
Geographic location	Shenzhen coastal area
Environment	Phycosphere environment
Biotic relationship	Related bacterium with algae
Temperature	10-40℃ (optimum at 30℃)
Salinity	10–50 % (optimum at 30 %)
pH value	3.5–11.0 (optimum at 8.5)
Genome size	4,230,919 bp
GC content %	44.0 %
Number of contigs	19
Total contig size	4,217,010 bp
Largest contig	103,047 bp
Protein coning genes	4610
tRNAs	86
rRNAs	30
Genes with a predicted function	25
Potential AI-2 (*LuxS*) encoding site	Contig 5
Encoding-AI-2 (*LuxS*) gene length	471 bp

Several previous studies demonstrated that *Bacillus* spp. were able to inhibit the growth of harmful algal blooms. The accumulation of reactive oxygen species (ROS) was considered to be a potential mechanism underlying these algicidal properties; this would disrupt membrane integrity and the synthesis of pigment, and ultimately kill algal cells [28]. Liao et al. [10,11] also confirmed that algicidal bacteria kill or lyse *C. pyrenoidosa* cells by inhibiting three antioxidases’ (superoxide dismutase, peroxide dismutase, and catalase) activities against ROS. Under this circumstance, the algae cells will lost the self-protection ability to resist ROS and halt grow or reproduce [29].

As for the bioactive compounds, Wu et al. [20] demonstrated that the algicidal compounds in *Bacillus* sp. strain S51107 were indole-3-carboxaldehyde and cyclo (Pro-Phe). At present, the causative substance(s) or mechanisms related to the algicidal effects of *B. subtilis* strain JA remain unclear. According to the viewpoint of Demuez et al. (2015), the majority of algicidal bacteria cause effect on the growth of algae by excreting algicidal compounds. In accordance with this theory, we speculated that *B. subtilis* strain JA may secrete an as yet unidentified compound that causes the lysis of algal cells. The possible algicidal compounds including hydrolase, surfactant, amino acid derivatives, polyketides, and fatty acids derivatives [8,30,31]. Future studies should now aim to further purify and identify algicidal compounds originating from *B. subtilis* strain JA.

### 3.2 AI-2 profile and possible regulation role in algicidal ability

**Fig. 3:**
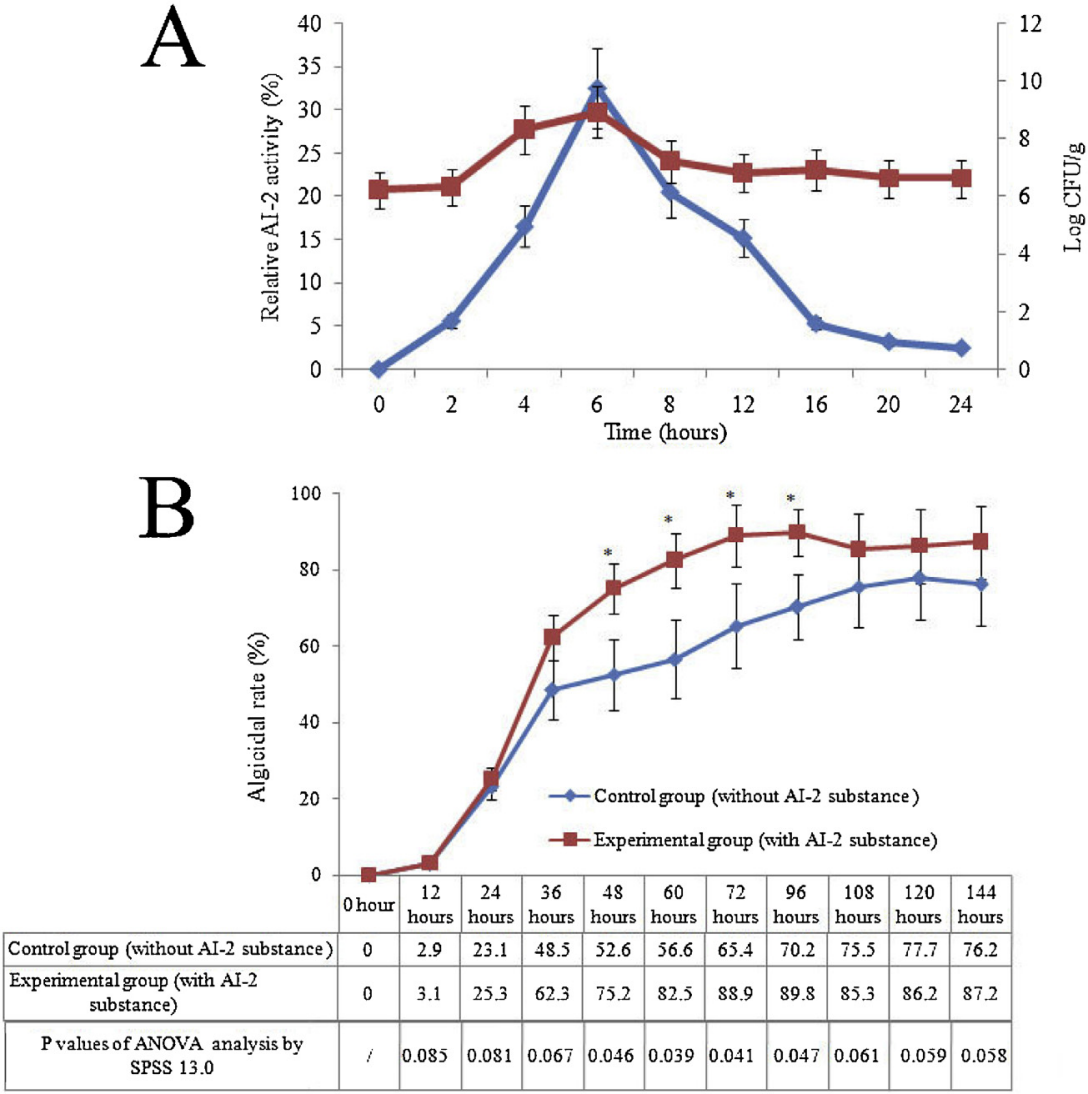
(A) Relative activity of AI-2 (red lines) and CFU counts (log CFU g^−1^, blue lines) of JA strain. Mean values of three independent experiments and standard deviations (the error bars) are indicated. (B) The algicidal rate of *B. subtilis* JA with or without QS substance. Data represent the mean + SD of triplicate measurements (n = 3) (For interpretation of the references to colour in this figure legend, the reader is referred to the web version of this article).

We used a reporter strain to detect the AI-2 QS system in order to verify the QS-synthesizing activity of *B. subtilis* JA. Our analysis detected AI-2 activity after 2 h incubation with strain JA, thus corresponding to the early exponential growth phase (Fig. 3A). Subsequently, levels of AI-2 levels increased with increasing biomass, reaching maximal levels during the late exponential or early stationary growth phase. To investigate whether the QS molecule had an essential role in algicidal ability, we carried out an additional experiment. As shown in Fig. 3B, the algicidal rate was markedly higher (increased by 23.4–47.6 %) in the presence of an oligopeptide than in the untreated group (P < 0.05). These results demonstrated that pretreatment with oligopeptide significantly improved the algicidal effects, thus suggesting potential roles for QS in both bacterial behavior and ecological response. It has been established that the algicidal activity of most reported bacteria are dependent on cell density and only exhibit algicidal activity at a certain density. Kang et al. (2015) further suggested that the threshold density (10^5^ cells/mL) of *Lactobacillus paraplantarum* might be a prerequisite for achieving the successful termination of natural *A. ﬂosaquae* blooms. Several previous studies have demonstrated that quorum sensing, mediated by homoserine lactone and alkylquinolone, are associated with the regulation of algicidal activity in Gram-negative bacteria [[32], [33], [34]]. The algicidal abilities of Gram-positive bacteria also depend on cell density [4]. Transcriptomic analysis has revealed that in *B. thuringiensis*, the NprR-NprX QS system controls the expression of at least 41 genes that encode neutral protease (NprA), degradative enzymes and other proteins; it is also involved in nutrient supply, stress and antibiotic resistance and controls the synthesis of kurstakin peptide. Our results provide further evidence that the algicidal activity may be regulated by QS signals. Furthermore, an improved understanding of the interaction between algae and bacteria is necessary before we can design and optimize strategies with which to control harmful algae blooms [35].

### 3.3 The genome features of *B. subtilis* JA

**Fig. 4:**
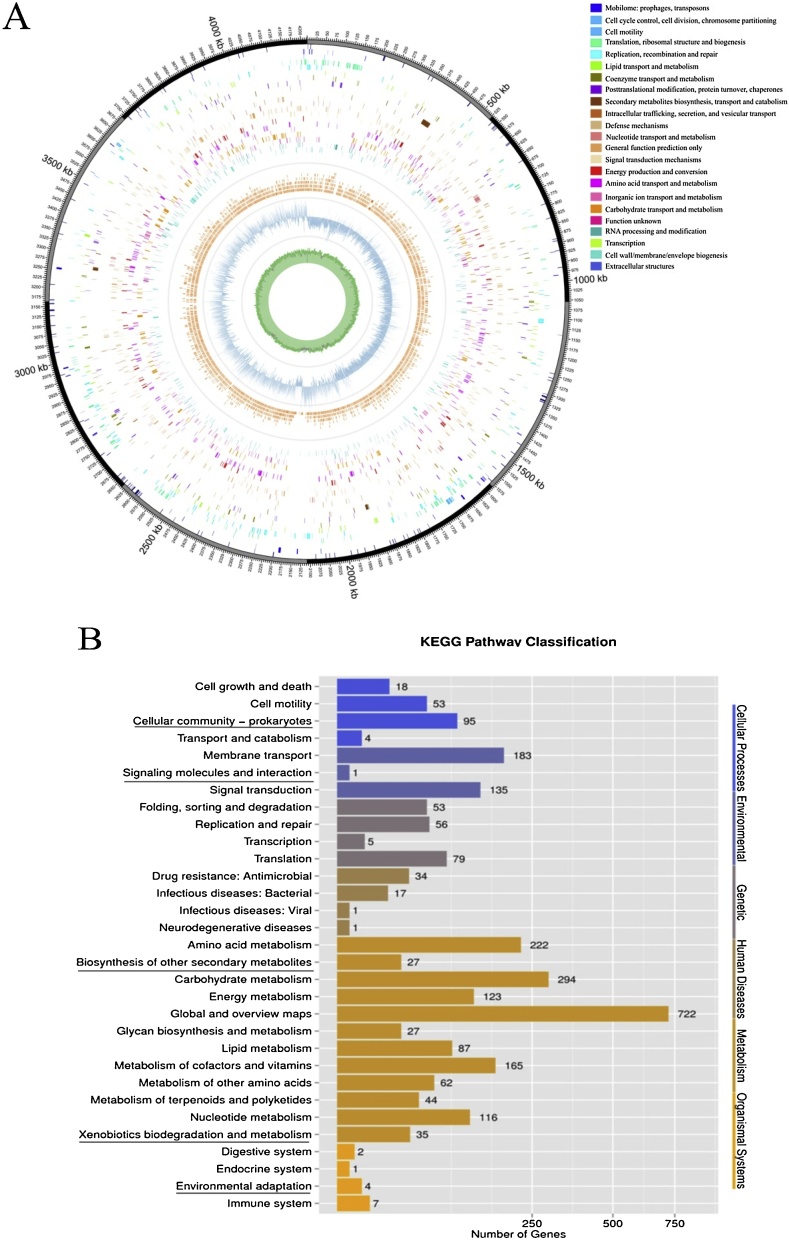
(A) Circular map for the whole genome of *B. subtilis* JA. From the outside to the center: encoding genes, predicted CDSs transcribed in the clockwise (or counter-clockwise) direction, ncRNA, GC percent (%), and GC skew (G + C/G-C) in a 1000-bp window. (B) Functional category distribution of *B. subtilis* JA (based on KEGG function statistics). The potential functions involved in algicidal active were marked with underlines.

The specific features of *B. subtilis* JA genome are summarized in Table 2 and Fig. 4 (a single circular chromosome). The entire genome of *B. subtilis* JA contained 4,230,919 bases and a G + C content of 44.0 %. There were 4,610 protein coding gene sequences with an mean molecular weight of 802.3 bp, as predicted by Glimmer version 3.02 (http://www.cbcb.umd.edu/sortware/glimmer), giving a coding intensity of 83.92 %. Genome assembly yielded 19 contigs with a total contig size of 3,698,805 bp. We also identified 221 non-coding RNAs in the genome, including 86 tRNAs, 30 rRNAs, and 95 other RNAs. Homologous comparison by BLAST revealed 2,673 CDS sequences comprising 25 functional COGs (http://www.ncbi.nim.nih.gov/COG/) and a portion of the CDS that included 31 Kyoto Encyclopedia of Genes and Genomes (KEGG) metabolic pathways. Of the coding sequences, the five most abundant groups in the category of molecular function were global & overview maps (722 genes), carbohydrate metabolism (294 genes), amino acid metabolism (222 genes), membrane transport (183 genes), cofactors and vitamins metabolism (165 genes) and signal transduction (135 genes), thus suggesting that proteins involved in cellular function and metabolism are responsible for maintaining the high metabolic activity of this bacterial species. In contrast, few genes were annotated in several other subgroups, including transcription activity, environmental adaptation, and transport ability, implying that fewer proteins encoded by this species would participate in such molecular processes. The final genome sequence was deposited in the NCBI database and the accession number is CP045425-CP045426.

The QS signaling pathway usually mediates inter-species communication by producing small extracellular signal molecules [36]. To investigate QS signaling, we performed *in silico* annotation to identify candidate AI-2 genes based on sequence BLAST analysis. One gene, encoding a putative *LuxS* gene, was subsequently found in contig 4 of the *B. subtilis* strain JA isolate. The bioinformatics analysis confirmed the presence of essential quorum sensing-related genes. This *LuxS*-encoding gene is 471 bp in molecular weight, thus suggesting its potential ability to manipulate cell density via QS signals [37]. A similar report indicated that *LuxS* is frequently detected in species that are closely related to algal-associated bacteria. Previous reports also indicate that the presence of *LuxI* in close vicinity is commonly detected in QS bacteria [38]. Based on comQXPA gene cluster is the main responser to AI-2 (or related genes) and involved in the biosynthesis of oligopeptide-type pheromone in *Bacillus* sp. [17]. We speculated that exist linkage between *LuxS* and pheromone encoding genes (such as ComX and Com Q), which supply a possible bridge to understand the modulate role of AI-2 to Gram-positive bacteria algicidal behavior. In addition, we identified multiple protease-coding genes downstream of *LuxS*; the proteins encoded by these genes are believed to contribute to the ability of *B. subtilis* strain JA to lyse its host. Previous research has reported that proteases represent candidate algicidal substances. For example, Aﬁ et al. (1996) reported that the degradation of *Chlorella vulgaris* by *P. oleovorans* and *Flavobacterium aquatile* was probably mediated by serine protease. Chen et al. (2011) further reported that l-amino acid oxidase from *Aquimarina* sp. exhibited algicidal activity against *M. aeruginosa.* Accordingly, this microorganism represents a potential algicidal bacterium with the ability to lyse algae. The whole-genome sequence generated in this study provides a deeper understanding of the relationships between bacteria and algae under the control of AHLs, and may facilitate the development of new microbial methods to control HABs.

## 4 Conclusions

In this work, we report the complete genome sequence of *B. subtilis* strain JA and describe its genomic features. In particular, we identified a potential AI-2 gene, *LuxS*, from this isolate and found that the antialgal activity of *B. subtilis* strain JA is likely regulated by a quorum sensing system. Although the specific substances responsible for this algicidal activity remain unidentified, our data improve our understanding of the interactions between dinoflagellates and indigenous algicidal bacteria through chemical signal molecules and may aid in the design and optimization of strategies with which to control harmful algae blooms.

## Author statement

SZ isolated the strains. XD and JZ performed the strain characterization including algicidal rate and quorum sensing production. CM and PZ performed the DNA isolation, genome sequencing, annotation and analyzes. JZ, SZ and PZ wrote the manuscript.

## Declaration of Competing Interest

There are no conﬂicts of interest to declare.
